# Podoplanin expression in oral potentially malignant disorders and oral squamous cell carcinoma

**DOI:** 10.4317/jced.54213

**Published:** 2017-12-01

**Authors:** Deepa A G, Bindu Janardanan-Nair, Varun B R

**Affiliations:** 1 Assistant Professor, Department of Oral and Maxillofacial Pathology , Sree Mookambika Institute of Dental Sciences, Kulasekharam, Tamil Nadu, India; 2 Consultant oral pathologist, Dr. Vivek’s Dental Clinic, Vazhuthacaud, Trivandrum, Kerala, India; 3 Associate Professor, Department of Oral and Maxillofacial Pathology PMS College of Dental Science and Research, Thiruvananthapuram, Kerala, India

## Abstract

**Background:**

Podoplanin is a type I transmembrane sialomucin-like glycoprotein that is specifically expressed in lymphatic endothelial cells. Studies have shown that assessment of podoplanin expression in the epithelial cells can be used to predict the malignant transformation of potentially malignant disorders and the metastatic tendency of primary head and neck squamous cell carcinoma. The aim of our study was to compare the expression of podoplanin in oral leukoplakia, oral submucous fibrosis and oral squamous cell carcinoma with that in normal buccal mucosa by immunohistochemical methods.

**Material and Methods:**

Immunohistochemical expression of podoplanin was analyzed in 20 cases each of oral leukoplakia, oral submucous fibrosis, oral squamous cell carcinoma and normal buccal mucosa, with monoclonal antibody D2-40. The expression of podoplanin was graded from grade 0-4.

**Results:**

There was a statistically significant upregulation of the grades of podoplanin expression in oral squamous cell carcinoma(100%), oral submucous fibrosis (90%) and oral leukoplakia (65%) when compared to that in normal mucosa(35%). Podoplanin expression increased with decrease in grades of differentiation in oral squamous cell carcinoma . Podoplanin expression in the samples of oral submucous fibrosis was higher than that in oral leukoplakia.

**Conclusions:**

Evaluation of podoplanin expression in the epithelial cells of oral dysplastic lesions may provide valuable information to predict their risk of malignant transformation.

** Key words:**Immunohistochemistry, Oral leukoplakia, Oral submucous fibrosis, Podoplanin, Squamous cell carcinoma.

## Introduction

Oral cancer is a predominant cause of cancer death, and oral squamous cell carcinoma (OSCC) accounts for more than 90% of all oral cancers (1). Early detection of potentially malignant disorders can decrease the morbidity and mortality associated with oral cancer. Currently, histologic assessment of epithelial dysplasia is the gold standard for determining the malignant transformation risk of oral leukoplakia. But, the accuracy of the histopathologic assessment of epithelial dysplasia depends on the quality of the tissue and the site at which a biopsy is taken. It also carries the risk of interobserver and intraobserver variability (2). The traditional grading system of oral epithelial dysplasia into mild, moderate and severe dysplasia does not permit accurate prediction of which cases may eventually develop into malignancy (3). Many investigators have remarked that potentially malignant disorders with epithelial dysplasia have shown to develop into cancer more readily than those without dysplasia (4,5). In contrast, all epithelial dysplasias do not develop into cancer and some have shown to regress with time (6,7). Oral cancer may also develop from lesions that lack dysplastic features (8).

Lack of objectivity in the histologic criteria such as the arbitrary division of gradings and difficulties in predicting the outcome of individual cases demanded the urgent identification of more reliable markers (9). One such marker is podoplanin which is widely used as a specific marker for lymphatic endothelial cells and lymphangiogenesis. Podoplanin is expressed on lymphatic but not on blood vessel endothelium (10).

Human podoplanin is a type I transmembrane sialomucin-like glycoprotein consisting of 162 amino acids. It is expressed in a wide variety of normal as well as tumor cells. In normal human tissue, podoplanin is expressed in kidney podocytes, skeletal muscle, placenta, lung, heart, myofibroblasts of the breast and salivary glands, osteoblasts and mesothelial cells. Podoplanin plays a significant role in preventing cellular adhesion and is involved in the regulation of the shape of podocyte foot processes and in the maintenance of glomerular permeability (10,11).

Various types of tumor cells also express podoplanin such as vascular tumors, central nervous system, germ cell tumors and squamous cell carcinoma. Podoplanin might favour tumor invasion through its ability to remodel actin in the cytoskeleton of tumor cells, resulting in increased motility. The association between podoplanin and the actin cytoskeleton seems to be mediated by ezrin, which is markedly phosphorylated in the presence of podoplanin overexpression. Podoplanin expression increases the activities of Rho GTPases, mainly Rho A, which might indicate a change in the cytoskeletal organization of the cells (12). Wicki and Christofori showed that invasion of podoplanin- expressing tumor cells was correlated with an overexpression of matrix metalloproteinases (MMPs) and it could be inhibited by specific inhibitors of MMPs (10).

In the past, podoplanin had been used often to assess intratumoral and peritumoral lymphovascular density in oral squamous cell carcinoma, which was correlated with metastatic spread to the lymph nodes and a poor prognosis (13-15). Studies have proven that podoplanin is also expressed in the epithelial cells of oral dysplastic and hyperplastic lesions with a risk of cancer development(8,16-21).

The purpose of our study was to compare the imunohistochemical expression of podoplanin in oral leukoplakia, oral submucous fibrosis(OSF) and OSCC with that in normal mucosa.

## Material and Methods

-Patients 

Formalin fixed paraffin embedded blocks were obtained from the archives of Department of Oral and Maxillofacial Pathology. Twenty cases each of leukoplakia, OSF and OSCC in the age group of 20-70 years were included in the study. Fresh H& E stai-ned sections were prepared from the archived blocks of leukoplakia, OSF and OSCC. Tissue sections without epithelium and tissues from non-oropharyngeal sites were excluded. Each section was re-examined by a single qualified oral pathologist to con-firm the histopathological diagnosis. Cases of leukoplakia were graded by the WHO criteria for dysplasia, OSF by classification of Pindborg JJ and Sirsat SM and OSCC by Broder’s classification(22,23). Control group consisted of twenty samples of normal non- inflamed buccal mucosa obtained during removal of impacted third molar. Demographic and clinical data of the patients are shown in  Table 1. The study was reviewed and approved by the Institutional Ethical Committee. Written informed consent was obtained from all patients.

**Table 1:**
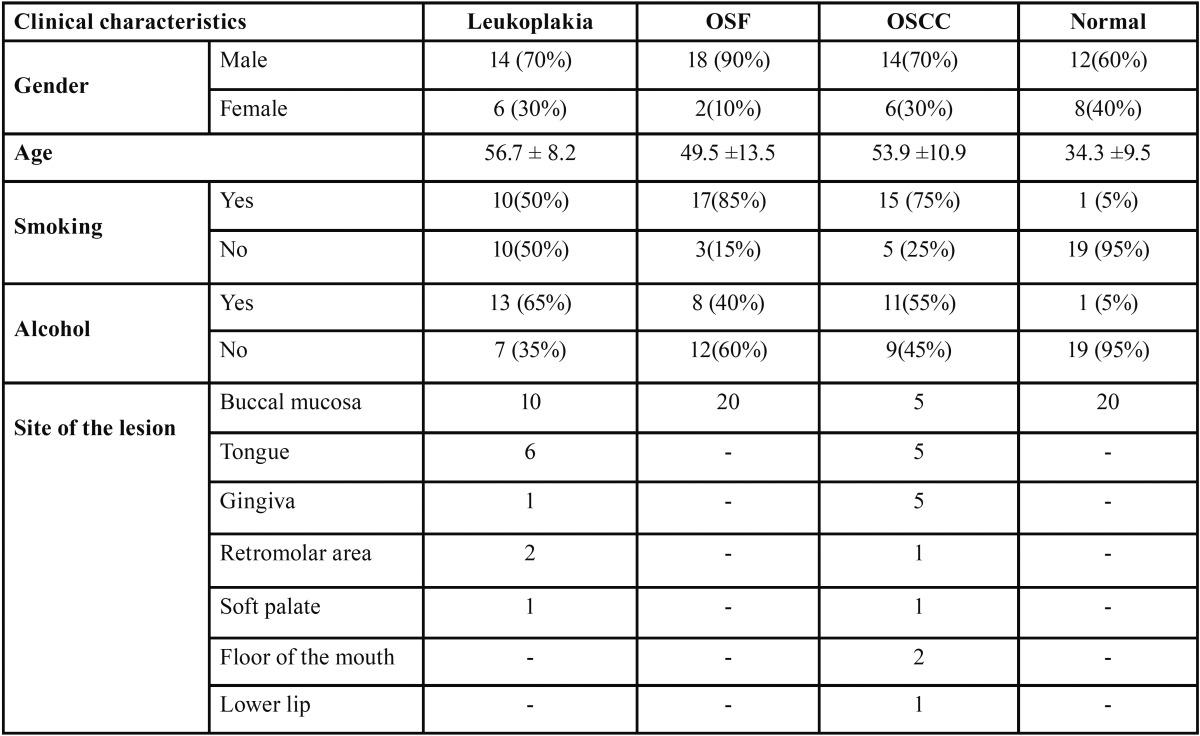
Demographic and clinical data.

-Immunohistochemistry procedure

Four micrometer thick sections from formalin fixed, paraffin embedded tissue blocks were mounted on positively charged 3-amino propyl triethoxy silane (APES) coated glass slides. Slides were deparaffinized in xylene and rehydrated through decreasig concentrations of ethanol. For antigen retrieval, slides were heated in a pressure cooker in TRIS- EDTA buffer for 5 minutes. Endogenous peroxidase activity was blocked with 3% hydrogen peroxide for 10 minutes and the slides were treated with Ultra violet block reagent for 5 minutes. Slides were incubated with mouse monoclonal antihuman podoplanin primary antibody (D2-40) (Dakocytomation, California Inc. USA) at room temperature for 1 hour, followed by secondary antibo dy horse radish peroxidase (Dakocytomation, California Inc. USA) for 30 minutes. Staining was visualised with 3’ –Diaminobenzidine Tetrahy-drochloride - a substrate chromogen (Dakocytomation, California Inc. USA) and counter stained with Harris hematoxylin. Sections were then dehydrated, cleared, mounted and observed under light microscope.

-Evaluation

Podoplanin expression was graded according to the procedure done by Inoue *et al.* (8). The score was based on the examination of the whole section by three oral pathologists who were blinded to the clinical information, using a multi headed microscope. Expression of podoplanin in the lymphatic endothelial cells in the connective tissue served as the positive control.

Grading of podoplanin expression 

Expression of podoplanin was predominantly membranous. Expression was graded as;

0- If no expression is observed in any part of the epithelium

1- If intensity of expression is weaker than that in lymphatic vessels and observed in the basal layer of epithelium.

2- If intensity of expression is weaker than that in lymphatic vessels and observed in more than two layers of epithelium.

3- If intensity of expression is equal to that in lymphatic vessels and observed in the basal layer of epithelium.

4- If intensity of expression is equal to that in lymphatic vessels and observed in more than two layers of epithelium.

-Statistical analysis:

Data was analyzed using computer software, Statistical Package for Social Sciences (SPSS) version 16 for Windows Operating System using Kruskal-Wallis and Mann-Whitney tests. The association between podoplanin expression status and clinicopathologic parameters were analyzed using the chi- squared test. A multivariate analysis was done using clinical data, histopathologic data, and podoplanin expression as cofactors to evaluate if podoplanin is an independent cofactor for cancer development.

## Results

-Podoplanin expression and clinicopathologic characteristics

Among the 20 cases of oral leukoplakia, 14 were males and 6 were females. The mean age of the patients was 56.7 ± 8.2 years. Most frequently affected sites were buccal mucosa (10 cases), followed by tongue (6 cases). Of the 20 cases of OSF, 18 were males and 2 were females. The average age of the patients was 49.5 ± 13.5 years. Out of the 20 cases of OSCC, 14 were males and 6 were females. Buccal mucosa, gingiva and lateral border of tongue were most commonly affected (5 cases each). Average age of the patients was 53.9 ± 10.9 years. There was no statistically significant correlation between podoplanin expression and age, gender or site of the lesion in any of the groups.

-Podoplanin expression in oral leukoplakia, OSF, OSCC and normal buccal mucosa

**Figure 1:**
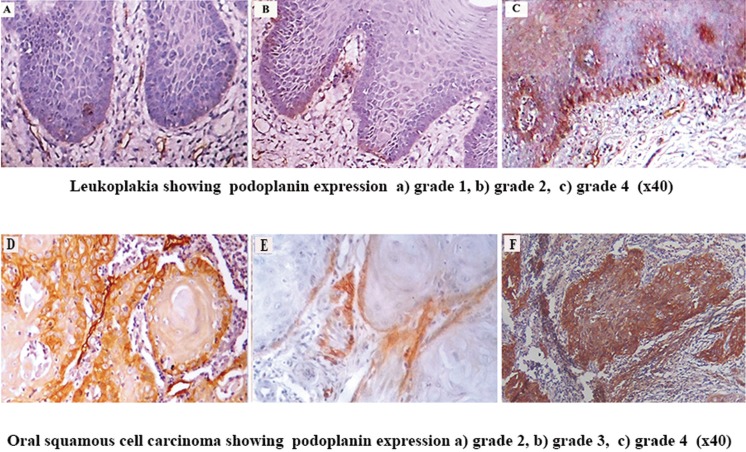
Leukoplakia showing podoplanin expression A) grade 1, B) grade 2, C) grade 4 (x40) Oral squamous cell carcinoma showing podoplanin expression D) grade 2, E) grade 3, F) grade 4 (x40).

**Figure 2:**
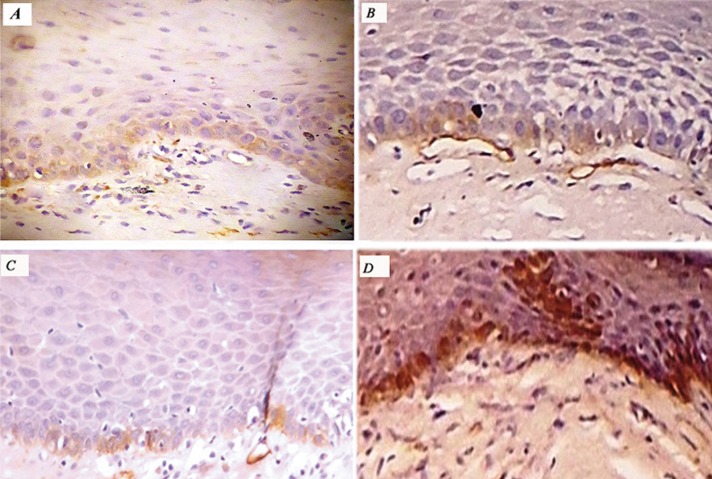
Oral submucous fibrosis showing podoplanin expression A) grade 1 B) grade 2, C) grade 3, D)grade 4 (x40).

**Figure 3:**
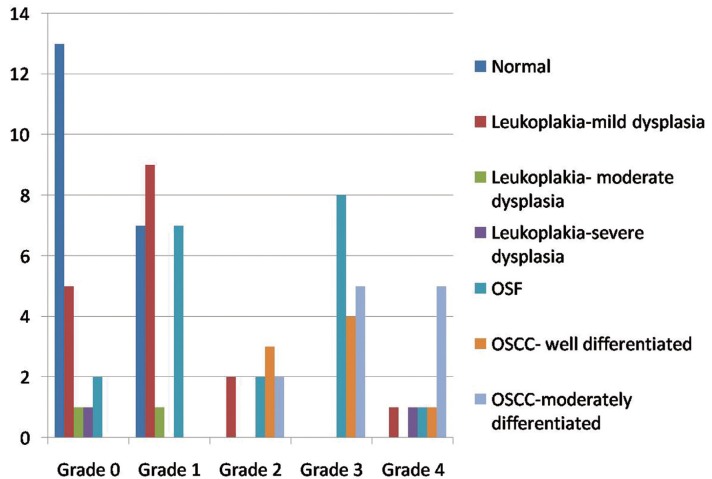
Graph showing the association between histologic grading and podoplanin expression.

Among the 20 cases of oral leukoplakia, 13 cases (65%) showed a positive expression of podoplanin in the epithelium, whereas 7 cases (35%) were negative. Among the 20 cases of OSF, 18 cases (90%) had positive expression of podoplanin; 2 cases (10%) showed grade 0. Among OSCC, all cases showed positive podoplanin expression. Among the 20 samples of normal mucosa, 13 (65%) were negative for podoplanin. Seven tissues (35%) showed positive podoplanin expression focally in small groups of cells in the basal layer of epithelium (grade 1). Podoplanin expression in oral leukoplakia and OSCC are shown in figure 1 whereas expression in OSF is given in figure 2. Graphical representation of podoplanin expression in normal mucosa, OSF, different histopathological grades of oral leukoplakia and OSCC are shown in figure 3.

Comparison of grades of podoplanin expression among oral leukoplakia, OSF and OSCC with that in normal mucosa using Kruskal-Wallis test showed that the variation in the grades of podoplanin expression among groups was significant at 0.000 level ( Table 2). Pair wise comparison of podoplanin expression using Mann-Whitney test showed that the grades of podoplanin expression is significantly high among OSCC (Z=5.4, *p*<0.01) and OSF (Z=3.69, *p*<0.01) when compared to that in normal oral mucosa. The associations between podoplanin expression status and clinicopathologic parameters were analyzed using the chi- squared test for categoric variables. When the multivariate analysis was done, a highly significant association (*p*< 0.001) was seen between podoplanin expression and grade of differentiation in normal mucosa versus OSCC.

**Table 2:**
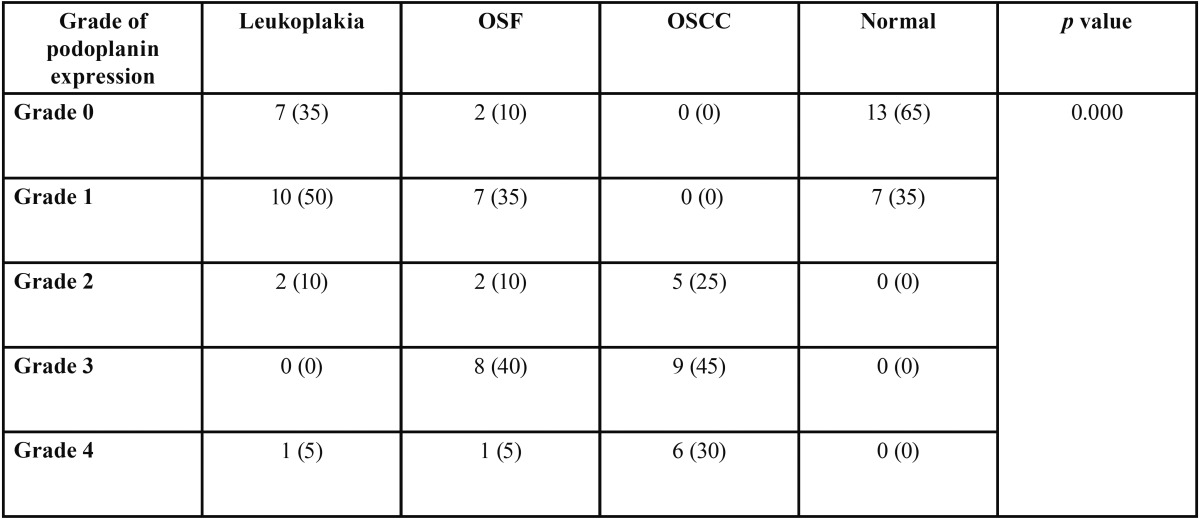
Comparison of podoplanin expression in oral squamous cell carcinoma,oral leukoplakia,oral submucous fibrosis and normal mucosa.

## Discussion

Podoplanin expression was frequently used for evaluating the lymphovascular density in head and neck squamous cell carcinoma (13,14). Yuan *et al.* first observed podoplanin expression in the basal cell layers in some of the hyperplastic and dysplastic epithelial areas adjacent to tumors (17). Kawaguchi *et al.* concluded that podoplanin expression extending to suprabasal layers in the epithelia of oral PMDs may represent upward clonal expansion of stem cells during carcinogenesis; and oral premalignancy with such clonal expansion may imply significantly higher risk of malignant transformation (16). Studies conducted by Inoue *et al.*, Funayama *et al.*, and de Vincent *et al.* also showed podoplanin expression in dysplastic cells of PMDs (8,18,21).

In the present study, we analyzed the immunohistochemical expression of podoplanin in 20 cases each of oral leukoplakia, OSF, OSCC and normal mucosa, and correlated it with clinicopathological parameters. Positive expression of podoplanin was observed in 65% of leukoplakia cases whereas 90% of OSF cases were podoplanin positive. Among OSCC, all cases showed positive podoplanin expression. Focal podoplanin positivity (grade 1) was expressed by 35% of normal mucosa. A statistically significant increase in podoplanin expression was observed in OSCC (Z=5.4, *p*<0.01) and OSF (Z=3.69, *p*<0.01) when compared to that in normal oral mucosa.

Kawaguchi *et al.*, Funayama *et al.*, Inoue *et al.*, Kreppel *et al.*, de Vincent *et al.* and Patil *et al.* showed that intensity of podopla-nin expression increased with the severity of dysplasia and suggested it as a useful predictor for the risk of cancer development in oral PMDs (8,16,18,20,21,24). In our study, podoplanin positivity was observed in 65% cases of leukoplakia, which was nearly similar to that of Funayama *et al.* As most of our samples were mild dysplasia (85%), we could not get a statistically significant correlation between severity of dysplasia and podoplanin expression.

Kawaguchi *et al.* observed that 37% of oral cancers developed in patients with podoplanin negative PMDs. They suggested that these podoplanin negative lesions in the study might have been biopsied before the abnormality developed or the biopsies might have been taken from a different clonal site other than the one in which cancer eventually developed (16). These factors may be attributed for the negative podoplanin expression in our study also.

Speight *et al.* have reported a higher malignant transformation rate of OSF than leukoplakia (25). In our study, among the 20 cases of OSF, 18 cases had positive expression of podoplanin; one was grade 4 and eight cases show ed grade 3. The expression of podoplanin in OSF was 90%; which was much higher than that in leukoplakia (65%), probably suggesting its increased risk of malignant transformation. However, these results remain inconclusive as our sample size was small and long term follow up was not done. Out of the 20 cases of OSF, 4 were early stage and 16 were advanced stage. We could not find any correlation between the podoplanin expression and the histological stages of OSF.

Various studies have shown high podoplanin positivity in OSCC, [Prasad B *et al.* (90%), Inoue *et al.* (95.7%), Kreppel *et al.* (84%) and Funayama *et al.* (100%)] (8,18,26,27). A statistically significant association between podoplanin expression and grades of differentiation was observed by Inoue *et al.* whose grading criteria we followed. Similar statistical correlation was also shown by Parasad *et al.* and Patil *et al.* (24,26). In our study, 12.5% of the well differentiated OSCC, showed grade 4; whereas 41.5% of moderately differentiated OSCC were grade 4. None of the cases showed grade 0 or grade 1. Even though the results were not statistically significant, an increasing expression of podoplanin with decreasing grades of differentiation was observed.

In our study, 35% samples showed positive podoplanin expression focally in small groups of cells in the basal layer of epithelium, which was similar to the observations by de Vincente *et al.* (21). Schacht *et al.* have reported focal expression of podoplanin in the basal keratinocytes of skin, cervix and esophagus (28). Contrary to these results, negative podoplanin expression was reported in oral epithelium by Funayama *et al.*, and Margaritescu *et al.* (8,29). Miyasaki *et al.* found positive podoplanin expression in the basal cells of inflamed gingival epithelium (30).

Inoue *et al.* have shown that increase in the podoplanin expression is associated with the development of dysplasia- carcinoma sequence in the oral cavity (8). In our study also, there was a statistically significant variation in the grades of podoplanin expression in OSCC (100%), OSF (90%) and oral leukoplakia (65%) when compared to that in normal mucosa (35%).

## Conclusions

At present, severity of epithelial dysplasia is the most important indicator for determining the risk of development of oral cancer in PMDs. Since the interpretation of podoplanin expression is relatively simple, it may also be included along with the standard histopathological examination of the dysplastic lesions.

A limitation of the present study is the small sample size. Further studies with larger sample size and follow up may elucidate the exact role of biomarkers like podoplanin in PMDs and invasive carcinoma.
